# Ultrawide Spectrum Metallic Plane Blackbody with Extremely High Absorption from 0.2 to 25 µm

**DOI:** 10.1002/advs.202411448

**Published:** 2024-11-21

**Authors:** Jin‐Yong Qi, Xue‐Qing Liu, Zi‐Jian Liu, Xin Zhang, Chao Li, Qi‐Dai Chen, Lei Wang, Hong‐Bo Sun

**Affiliations:** ^1^ State Key Laboratory of Integrated Optoelectronics College of Electronic Science and Engineering Jilin University Changchun 130012 China; ^2^ State Key Laboratory of Precision Measurement Technology and Instruments Department of Precision Instrument Tsinghua University Beijing 100084 China

**Keywords:** ultrawide spectrum, plane blackbody, high absorption, “V”‐ scanning, nonmaterial selectivity

## Abstract

A plane blackbody serves as a standard radiation source, providing a precise quantitative relationship between input radiation and the output of infrared detectors, which is essential component of space infrared remote sensing instruments. However, current plane blackbodies fabricated by coating or surface structuring are unable to achieve uniform and stable high absorption in the ultrawide spectral range spanning the UV‐VIS‐NIR‐MIR. Here, a femtosecond laser “V”‐ scanning method is proposed for the fabrication of cross‐scale multi‐layered micro‐ and nanocomposite structures on copper surfaces to realize ultrawide spectrum metallic plane blackbody with high absorption. The structures consist of a micrometer cone‐tip structure with a depth‐to‐width ratio of 7:1 (period 30 µm, depth 210 µm), an oxide layer with a thickness of more than 2 µm, and nanoparticles of different sizes, achieving uniform high absorption rates exceeding 99% in the localized spectral range of 400–700 nm and over 98% from the UV to MIR range of 200 nm to 25 µm. This strategy offers a generalized approach to enhance surface light absorption, with significant application potential in infrared calibration, passive radiation cooling, and stray light suppression.

## Graphical abstract

A universal femtosecond laser “V”‐ scanning method is proposed, and guided by pre‐theoretical simulations, a micro‐ and nanocomposite structure with a depth‐to‐width ratio of 7:1 is successfully fabricated on the copper surface, which achieves a light absorption of more than 99% in the localized (400–700 nm) and more than 98% in the UV to MIR (200 nm–25 µm).

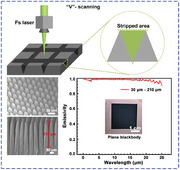

## 1 Introduction

Ultrawide spectrum metallic plane blackbody with excellent electromagnetic absorption properties has received significant attention in infrared calibration for meteorological monitoring, earth observation, military operations, and other fields.^[^ ^1^, ^2^, ^3^, ^4^, ^5^ ^]^ However, all infrared detection instruments need to be constantly calibrated using a blackbody radiation source before they can be used. According to Kirchhoff's law, a blackbody exhibits the strongest radiation capacity at any temperature and wavelength, absorbing all incident thermal radiation without reflection or transmission. It allows for the precise establishment of a quantitative relationship between input radiation and the output of the infrared (IR) detector, making blackbodies standard radiation sources for radiometric calibration of IR detectors.^[^ ^6^, ^7^, ^8^ ^]^ Therefore, development of plane blackbody with high radiation coefficients, high stability, and light weight is decisive for the high‐precision calibration of infrared detectors, and has become the inevitable trend of the development of infrared remote sensing technology in the future. Currently, to increase the surface absorptivity and/or decrease the reflectivity are the common routines for plane blackbodies, e.g., surface coatings and creating micro‐ and nanostructures on metallic surfaces. The coating method mainly involves spraying a high emissivity coating on the substrate surface, thereby changing the surface properties of the substrate. The basic principle of the coating method is the interferential phase elimination of materials with different refractive indices or the resonant absorption of oxidized nanoparticles, and the method is relatively simple, but has some disadvantages, such as long curing time, volatile, poor adhesion, not resistant to strong radiation, and poor high and low temperature stability.^[^ ^9^, ^10^, ^11^ ^]^ Alternatively, composite nanostructures covering on the surface of microstructures have emerged as strong candidates by increasing the absorptivity and decreasing the surface reflections.^[^ ^12^, ^13^, ^14^ ^]^ Due to the size effect or oxidation, the nanostructures on the surface enhance the absorption rate. Meanwhile, the high‐aspect‐ratio microstructure can confine within their interior, increasing the effective surface area or enhancing the resonance, collectively reducing light reflections. The higher the aspect ratio, the stronger the light‐trapping capability. It was virtually impossible for any technology to fabricate such a complex structure over a large area till the discovery of femtosecond laser‐induced micro‐nanostructures. Examples include micro‐ and nanofabrication techniques such as photolithography and nanoimprinting, which can easily create precise nanostructures that produce high light absorption at designed wavelengths. However, these techniques are expensive and not suitable for large‐area processing, industrialization, and high‐volume production.^[^ ^15^, ^16^, ^17^ ^]^

In contrast, femtosecond laser processing can be applied to any material due to the transient strong field effect. And it can produce large‐area samples by high‐speed scanning in a non‐vacuum environment.^[^ ^18^, ^19^, ^20^, ^21^, ^22^ ^]^ In this area, composite micro‐nanostructured silicon and metals emerged by high‐speed femtosecond laser scanning. They are famously termed as black silicon and color metal, respectively, and has paved the possible way for the realization of ideal plane blackbody of high broadband absorption in ultra‐wide spectrum. So far, although micro‐ and nanocomposite structures obtained by femtosecond laser processing on the surface of metallic materials have shown some broadband absorption properties,^[^ ^23^, ^24^, ^25^, ^26^, ^27^, ^28^, ^29^, ^30^, ^31^ ^]^ ultrawide spectrum metallic plane blackbodies with high absorption have not been achieved. Table S1 (Supporting Information) summarizes current work on metal blackbodies on copper, aluminum alloy, titanium alloy, and non‐metallic materials like silicon.^[^ ^24^, ^25^, ^26^, ^27^, ^28^, ^29^, ^30^, ^31^, ^32^ ^]^ For example, Zhong et al. fabricated nanoparticles and microparticles of different sizes on copper surfaces using ultrafast laser, which can modulate the light absorption capacity of copper surfaces between 10% and 90%.^[^ ^23^ ^]^ Fan et al. presented 97.3% and 94% light absorption in the VIS (400–750 nm) and NIR (1000–1800 nm) regions by ultrafast laser modification of micro‐ and nanoparticles on the metal surface, respectively.^[^ ^24^ ^]^ To further reduce the surface reflection, femtosecond laser composite processing method have been proposed. For example, Fan et al. pre‐introduced precursor micro‐nanostructures on the surface of copper by ultrafast laser, and then continued to grow nanowires on the surface of the micro‐nanostructures by thermal oxidation to enhance the light absorption, and finally achieved 97% light absorption in the range of 14–18 µm.^[^ ^26^ ^]^ Jiang et al. achieved excellent anti‐reflective properties by femtosecond laser pretreatment followed by chemical oxidation. There was an increase in absorption in both the UV‐IR spectral range, but the peak absorbance in the VIS and NIR spectral ranges only reached 96% and 97%, respectively.^[^ ^27^ ^]^ Most of these works can only achieve relatively high light absorption in a narrow bandwidth range, and these works provide the basis for the realization of an ideal blackbody. However, it remains challenging to determine the size and morphology of micro‐nanostructures, as well as their composite modes. Consequently, achieving a blackbody with an absorption rate exceeding 95% across the UV to infrared wavelength has become highly demanding. Among all the influencing factors, to obtain high‐aspect‐ratio conical structures on opaque metals poses the greatest challenge for the realization of broad‐spectrum, high emissivity plane blackbodies.

Here, since high aspect ratio structures cannot be realized by the conventional laser scanning process, we proposed a “V”‐ scanning method which can better modulate the morphology of cross‐scale multi‐layered micro‐ and nanocomposite structures. The method opened the processing window and succeeded to obtain high‐aspect‐ratio micrometer cone‐tip structures with period of 30 µm and 210 µm deep. Meanwhile, the microstructures were uniformly coated with an oxide layer over 2 µm thick and nanoparticles with different sizes. Accordingly, high absorption in ultra‐wide spectrum is steadily achieved through the geometrical light trapping effect of the micrometer structure, optical coupling absorption and the effective medium effect of the oxide and nanoparticle layers. In result, uniform high absorption > 99% in the localized spectral range of 400–700 nm and > 98% from the UV to MIR range of 200 nm to 25 µm were demonstrated theoretically and experimentally. The proposed method is applicable to various metallic and some non‐metallic materials, stability is the outstanding advantage of the method, its ultra‐broad spectrum is suitable for different application requirements, and it offers a general approach for infrared calibration, passive radiation cooling, and stray light suppression.

## 2 Results and Discussion

### 2.1 Fabrication of Composite Structures and Its Light Absorption Principle

**Figure 1:**
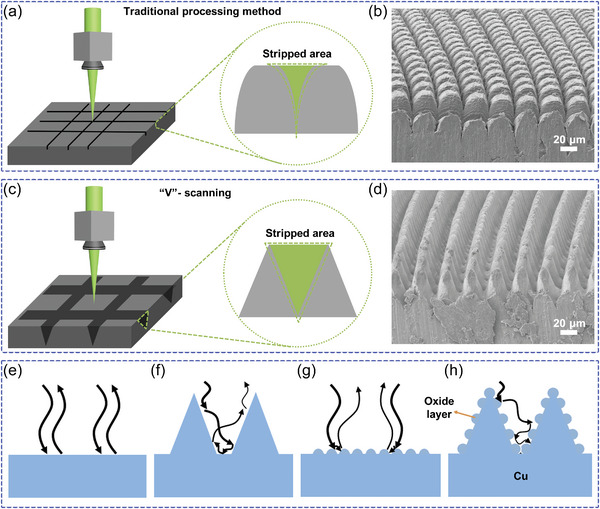
Fabrication of micro‐ and nanocomposite structures and its light absorption principle. a,b) Schematic diagrams of the conventional linear scanning method and its structural cross‐section. c,d) Schematic diagrams of the “V”‐ scanning method and its structural cross‐section. e–h) Light absorption principle of each component of micro‐ and nanocomposite structures.

According to Kirchhoff's law, the absorptivity is equal to the emissivity, which is also equal to 1‐reflectivity. In this paper, the emissivity is uniformly used to characterize the light absorption effect of the proposed micro‐ and nanocomposite structures.^[^ ^33^, ^34^ ^]^ **Figure** **1** shows the processing of the constructed micro‐nanocomposite structure and its light absorption principle. First, the different results of the conventional laser linear scanning method and the proposed “V”‐ scanning method are compared as shown in Figure 1a,c. In the conventional linear scanning method, the laser first ablates the material, and some of the spattered debris is subsequently deposited near the laser trajectory. Primary micro‐ and nano‐composite structures can be processed by setting the laser trajectory to a grid type. That is the laser at the same Y‐axis position by changing the focusing spot in the X‐axis at different positions to form a scanning line, with the number of laser scanning or scanning speed parameter changes, the depth of the scanning line can be adjusted within a limited range. In conjunction with the change in the Z‐axis, the laser focusing spot can penetrate deeper into the structure, the cross‐section of which is shown in the inset of Figure 1a, and the closer to the surface of the sample, the wider the groove obtained by laser processing, and the further away from the surface of the sample, the narrower the groove becomes due to the accumulation of debris. Then by changing the Y‐axis position for linear scanning in the X‐axis, a grating structure with a certain period is formed in the Y‐direction, and finally, by repeating the above steps for linear scanning in the Y‐axis, a primary grid‐type micro‐nanocomposite structure can be created. The structure prepared by this method is shown in Figure 1b, and due to the inability of this method to better regulate the period and depth of the prepared structure, it can be observed that the cross‐section of the single mini‐structures presents an approximately rectangular shape. Different cross‐sectional morphologies prepared by the conventional linear scanning method when the scanning line period is different are shown in Figure S1 (Supporting Information). As shown in Figure S1a (Supporting Information), when the scanning period is large, there is no interaction between the top of the individual rectangular structure and the laser, so a square unabated area appears at the top, and the cross‐section is shown in Figure S1b (Supporting Information). When the scanning period is reduced, the top unabated area of the individual rectangular structure is also reduced, as shown in Figure S1c,d (Supporting Information). Micro‐ and nanocomposite structures resembling cone tips are obtained only when the scanning period matches the spot size of the currently used processing system, as shown in Figure S1e,f (Supporting Information).

The conventional linear scanning method is also not conducive to structural depth modulation, as shown in Figure S2 (Supporting Information).In general, for the same laser energy, the structural depth increases as the number of laser scans increases or the laser scanning speed decreases. However, since the laser does not change the position of the X‐axis (in the case of Y‐axis linear scanning, and the same in the case of X‐axis linear scanning) during in‐linear scanning, even if the number of scans is increased or the scanning speed is decreased all the time, the depth inside the groove cannot be increased continuously due to the buildup of debris, as shown in Figure S2a (Supporting Information). As shown in Figure S2b (Supporting Information), the cross‐sectional morphology of the groove fabricated at different scanning speeds is demonstrated, and four scanning speeds (5, 10, 50, 100 mm s^−1^) are selected and shown in enlarged images, as in Figure S2c–f (Supporting Information). The cross‐sectional morphology was measured with a laser scanning confocal microscope, as in Figure S2g (Supporting Information), where it can be more clearly observed that the depth increases with decreasing velocity, but there is a limitation to the depth increase. In conclusion, the conventional linear scanning method cannot achieve a good modulation of the morphology of the structure, such as the depth‐to‐width ratio, so it does not realize the optimal light absorption effect.

Figure 1c shows the proposed method of “V”‐ scanning, by changing one line of the traditional grid‐type scanning to one face, i.e., by scanning several times at different positions in the X‐axis (Y‐axis linear scanning), together with the modulation of the Z‐axis, the final cross‐sectional morphology of the structure is obtained as shown in the inset of Figure 1c. Figure S3 (Supporting Information) gives details of the “V”‐ scanning, namely the changes in the removed area of the cross‐section for different number of scans. As shown in Figure S3 (Supporting Information), Figure S3a (Supporting Information) is a general overview of the machining process, and Figure S3b–f (Supporting Information) are schematic diagrams of the removed area of the cross‐section at different numbers of scans, respectively. As in Figure S3b (Supporting Information), in the first scan, the surface removed a wider area (dark gray). As in Figure S3c (Supporting Information), in the second scan, the z‐axis decreases by a certain height while decreasing the width of the scanned region. Similarly, with the increase of the number of scans, the removed area gradually penetrates deeper into the structure, and a cone‐tip structure with high depth‐to‐width ratio can be prepared. Unlike conventional linear scanning methods, this “V”‐ scanning method can control the laser removal area to present an inverted triangle in the cross‐section. As shown in Figure 1d, the final grid‐type structure obtained is the optimized micro‐ and nanocomposite structure due to the increased removal area, which is more conducive to debris removal inside the groove. The cross‐section of the structure presents an approximate conical shape instead of a rectangular shape. It is worth mentioning that although the method increases the scanning trajectory of the laser, the same or even deeper depth as the traditional low‐speed linear scanning method can be achieved by scanning multiple times at high speed at the same time. Considering the processing time, this paper achieves fast scanning of the laser by improving the scanning method using a galvanometer. In practical applications, the processing efficiency can be further improved by methods such as laser beam splitting.

Finally, as shown in Figure 1e–g, the principle of reflection reduction for each part of the micro‐ and nanocomposite structure is compared. As in Figure 1e, for a smooth metal surface, the incident light has a high reflection effect. As shown in Figure 1f, the micrometer‐sized cone‐tip structure acts as a geometrical trap for light, and according to theoretical expectation, the light absorption effect of the cone‐tip structure increases with depth at the same period. Therefore, as shown in Figure 1a, the traditional linear scanning method cannot better control the morphology of the structure such as depth‐to‐width ratio, and it cannot achieve a better light absorption effect. As in Figure 1g, the nanoparticles on the surface of the structure can act as an effective medium, which can effectively reduce reflections by mitigating the difference in refractive indices between the metal and free space. Finally, as shown in Figure 1h, the proposed “V”‐ scanning method can realize a micro‐ and nanocomposite structure with adjustable depth‐to‐width ratio on a metal surface: including a micrometer cone‐tip structure, an oxide layer and a nanoparticle layer. The ultra‐wide spectrum high absorption effect of this micro‐nanocomposite structure is achieved by the optimized geometrical light trapping effect of the micrometer structure, optical coupling absorption and the effective medium effect of the oxide layer and the nanoparticle layer.

### 2.2 Theoretical Verification

**Figure 2:**
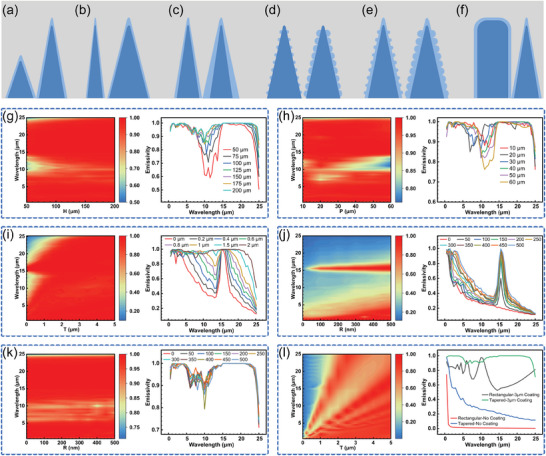
Theoretical simulation of the light absorption effect with different structural parameters. a–f) Schematic diagrams comparing the different parameters of the structure, respectively: a) height of the cone‐tip structure; b) period of the cone‐tip structure; c) thickness of the oxide layer; d) size of the particulate structure without the oxide layer; e) the size of the particulate structure with the oxide layer and f) the rectangular structure versus the cone‐tip structure; g–l) corresponds to a–f for the effect of light absorption when varying different parameters.

As shown in **Figure** **2**, the necessity of the proposed “V”‐ scanning method was verified using Comsol Multiphysics multiphysics field simulation software and further guided the fabrication of the subsequent structures. As shown in Figure 2a–f, six different structural morphologies were constructed using the control variable method by varying only one parameter of the structure at a time, namely, different heights (*H*) in Figure 2a, different periods (*P*) in Figure 2b, different thicknesses of the oxide layer (*T*) in Figure 2c, different particle sizes (*R*) without the oxide layer in Figure 2d, different particle sizes with the oxide layer in Figure 2e, and the comparison of the rectangular and cone‐tip structures in Figure 2f. Figure 2g–l shows the simulation results for these six different structural parameters, respectively, and each simulation consists of a graph of the emissivity distribution and a graph of the emissivity profile for typical parameter values. As in Figure 2g, by changing only the height of the cone‐tip structure, it can be seen from the emissivity distribution graph that with the increase of the height (horizontal coordinate), the emissivities at different wavelengths (vertical coordinate) are increased, and it can be more clearly observed from its profile that the higher the height, the higher the structural emissivity. Figure 2h compares the effect of different periods on the emissivity of the structure, and it can be observed that the structure has the highest emissivity at a period of about 30 µm. As shown in Figure 2i, different oxide thicknesses play a more critical role, comparing oxide thickness 0 and oxide thickness 0.2 µm, it can be observed that there is a significant improvement in the full‐band emissivity of the structure even if only 200 nm of oxide layer is added. The full‐band emissivity of the structure increases as the thickness of the oxide layer increases, but the effect of the oxide layer on increasing the emissivity of the structure essentially reaches its limit when the thickness of the oxide layer reaches approximately 2 µm.

As in Figure 2j, the effect of different nanoparticle radii on the structural emissivity in the absence of an oxide layer is compared. Since nanoparticles and oxide layers have similar resonant light absorption and effective medium effects, the structural emissivity increases with increasing nanoparticles. As in Figure 2k, the effect of different nanoparticle radii on the emissivity is compared for an oxide layer thickness of 2 µm, and it can be observed that at this point the structural emissivity remains essentially unchanged as the nanoparticle radius changes, confirming that the oxide layer and the nanoparticle layer play similar roles in increasing the structural emissivity. As in Figure 2l, the distribution of emissivity with the thickness of the oxide layer is finally compared between the structure with a rectangular cross‐section obtained by the conventional linear scanning method and the cone‐tip structure obtained by the “V”‐ scanning method. The emissivity curves of the two structures are blue and red for no oxide layer, and green and black for an oxide layer thickness of 3 µm. It is observed that the emissivity response of the cone‐tip structure is better than that of the rectangular structure regardless of the presence or absence of the oxide layer. Here, the necessity of the proposed “V”‐ scanning method is verified by theoretical simulations, by which the morphology of the micro‐ and nanocomposite structures can be better regulated to achieve optimal light absorption.

### 2.3 Element Characterization

**Figure 3:**
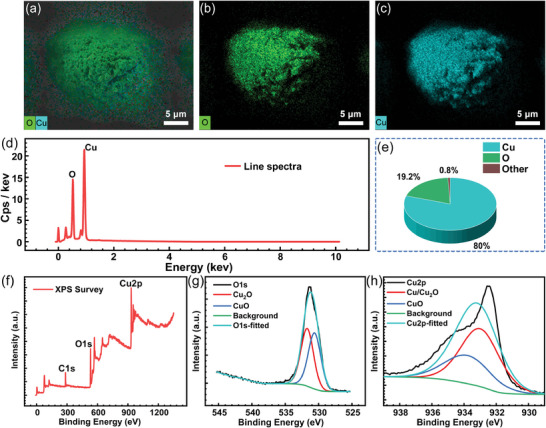
Characterization of the structural surface element distribution by EDS and XPS. a–c) Face scans of the structural surface elements of a single small cone‐tip as well as the respective face scans of the O and Cu elements. d) Line total spectra of the structural surface element distribution. e) Pie chart of the structural surface element distribution. f) Full spectra obtained by XPS characterization. g,h) Test results of the O 1s orbitals and the Cu 2p orbitals.

As in **Figure** **3**, the surface element distribution of the fabricated micro‐ and nanocomposite structures was first characterized by EDS. As shown in Figure 3a, which demonstrates the total elemental distribution on the surface of a single small cone‐tip structure, it can be observed that the surface is predominantly covered by a large amount of O and Cu, and the face‐scan maps of each of the two elements, O and Cu, are shown in Figure 3b,c. Next, Figure 3d gives a line total spectrum of the elemental distribution on the surface of the structure, and it can be observed that the Cu and O elemental peaks are much higher than the other trace elements. Figure 3e presents a pie chart of the elemental content on the surface of the structure, and it can be observed that the percentage of Cu element reaches 80% and O element reaches 19.2%, so the surface of the fabricated micro‐ and nano‐cone‐tip structure is covered with an oxide layer. Next, the composition of the oxide layer on the surface of the structure was specifically analyzed by XPS, and the results are shown in Figure 3f–h. The full spectrum obtained from the XPS test is given in Figure 3f, which once again corroborates the presence of large amounts of Cu and O elements on the surface of the structure. The test results for O 1s orbitals and Cu 2p orbitals are given in Figure 3g,f, respectively, and from the fitting results, it can be observed that the O elements on the surface of the structure are mainly in the form of CuO and Cu_2_O.^[^ ^35^, ^36^, ^37^ ^]^

### 2.4 Structural Morphology Control and Emissivity Measurements

**Figure 4:**
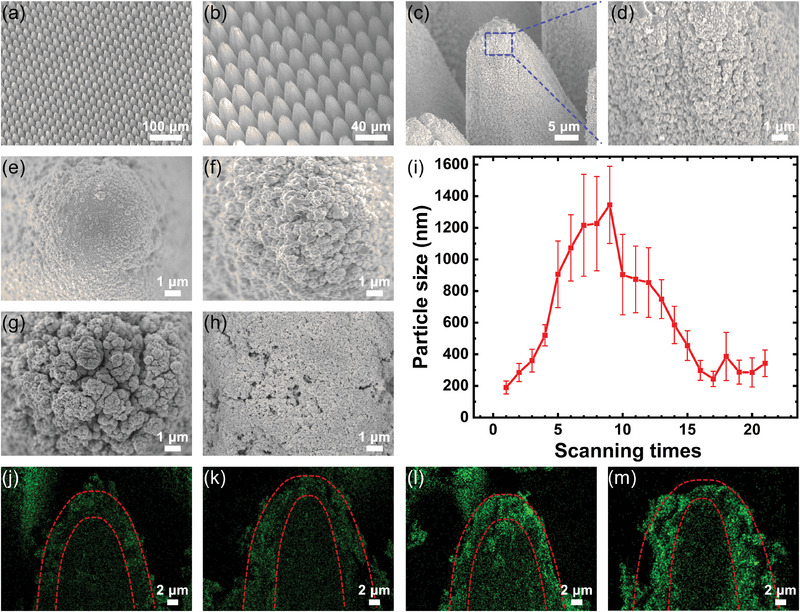
Modulation of structural nanoparticle size and oxide layer thickness. a–d) Top views at different magnifications. e–h) SEM images of the structures fabricated at pulse energy of 0.6, 1.7, 5.6, and 15.3 µJ. The scanning times were 5 times. i) The change in nanoparticle diameter for the number of scans from 1 to 21 with a pulse energy of 1.7 µJ. j–m) Elemental distributions of structural cross sections corresponding to e–h.

The theoretical simulations answered what kind of structure can achieve the best broad‐spectrum light absorption, and the proposed micro‐ and nanocomposite structures were next fabricated by “V”‐ scanning method. As shown in **Figure** **4**, firstly, top views of the fabricated micro‐nanocomposite structures with different magnifications are shown in Figure 4a–d. It can be observed that the structure morphology is homogeneous and the surface of the structure is covered with a layer of nanoparticles. Figure 4e–h demonstrates that the nanoparticles change from small to large and then to small again as the degree of interaction between the laser and the surface of the structure (e.g., pulse energy, scanning times) deepens. Taking the laser energy as an example, the diameter of nanoparticles on the surface of the micrometer cone‐tip structure is also smaller when the laser energy is smaller, as in Figure 4e. As the laser energy increases, the diameter of the nanoparticles also increases as shown in Figure 4f,g. As shown in Figure 4h, when the laser energy is too high, the surface of the micrometer cone‐tip structure is excessively ablated, deposited, and melted, and the nanoparticle diameter decreases instead. In addition, the size of nanoparticles shows a similar trend as the number of scans increases, as shown in Figure 4i. The statistical method is shown in Figure 4c,d, the same regions of micrometer cone‐tip structures under different laser parameters were selected, and statistical methods were used to count the sizes of nanoparticles on their surfaces through their electron microscopy maps at larger magnifications. Based on the statistical results, the dependence relationship between the laser parameters and the size of nanoparticles is established, to realize the regulation of the size of nanoparticles. In addition to the nanoparticle diameter, the gaps between the nanoparticles also affect the absorption rate of the structure.^[^ ^38^, ^39^ ^]^ The nanoparticles in this paper act as a similar effect to the oxide layer and the effect of nanoparticle porosity is no longer specifically discussed. Finally, the cross‐sectional EDS elemental characterization results for the structures shown in Figure 4e–h are presented in Figure 4j–m, where it can be observed that the thickness of the oxide layer formed by ablation is greater than 2 µm, regardless of the degree of interaction between the laser and the surface of the structure. The cross‐section of the cone‐tip structure was dissected using a focused ion beam (FIB), and the oxide and substrate layers can be clearly distinguished from each other by the SEM cross‐section image. The corresponding test results are placed in Figure S4 (Supporting Information), where the red dashed line helps to better distinguish the oxide layer from the substrate layer. From Figure S4 (Supporting Information), it can be found that the thickness of the oxide layer formed by ablation exceeds 2 µm, and this conclusion is consistent with that of Figure 4j–m. According to theoretical simulations, the effect of enhanced light absorption provided by the oxide layer and nanoparticle layer of the micro‐ and nanocomposite structure reaches the limit when the thickness of the oxide layer exceeds 2 µm. Therefore, the proposed “V”‐ scanning method can achieve the best broad‐spectrum light absorption effect in the modulation of oxide layer thickness and nanoparticles.

**Figure 5:**
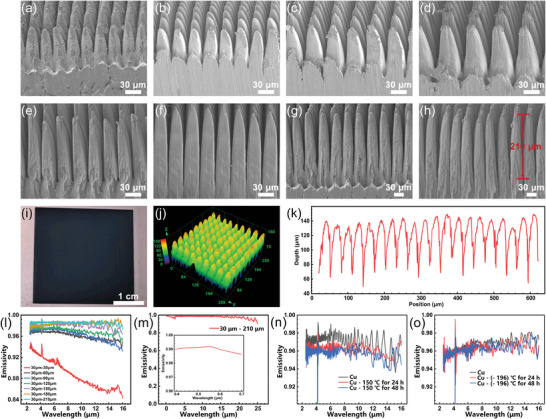
Modulation of micrometer cone‐tip structures and its emissivity results test. a–d) Periods of different cone‐tip structures: 20, 30, 40, 50 µm. e–h) Different heights of the cone‐tip structures when the periods are all 30 µm: 120 µm, 150 µm, 180 µm, 210 µm. i) Physical drawings of copper used for emissivity test. j,k) 3D morphology and cross‐section curves of the structures shown in i. l) Emissivity test results of the structures with different depth‐to‐width ratios. m) Full‐spectrum (200 nm–25 µm) test results for a structure with a depth‐to‐width ratio of 7:1 and test results for 400—700 nm (inset). n,o) High and low‐temperature stability tests of the structures.

After modulating the oxide and nanoparticle layers of the proposed micro‐ and nanocomposite structures, the depth‐to‐width ratio of the micrometer cone‐tip structure is the key factor that continues to enhance light absorption. According to theoretical simulations, the micrometer cone‐tip structure possesses the best light absorption effect at a period of about 30 µm, but it is a challenge to achieve a height of tens or even hundreds of micrometers at a period of 30 µm. The “V”‐ scanning method proposed in this paper is a great solution to this problem, which can better remove the debris when the depth of the micrometer cone‐tip structure is deeper, which is conducive to the depth enhancement of the micrometer cone‐tip structure, and it can well regulate the period and other morphology of the cone‐tip structure, the corresponding structural regulation ability is shown in **Figure** **5**. Figure 5a–d demonstrates the modulation of the period of the micrometer cone‐tip structure by the “V”‐ scanning method, with the periods ranging from small to large as 20, 30, 40, and 50 µm, respectively, which illustrates the flexibility of the method to modulate the period of the micrometer cone‐tip structure. Fixed the period of the micrometer cone‐tip structure is 30 µm, and Figure 5e–h demonstrates the heights of different micrometer cone‐tip structures with the same period, with heights of 120, 150, 180, and 210 µm, i.e., the cone‐tip structure depth‐to‐width ratios are 4:1, 5:1, 6:1, and 7:1, respectively. Figure 5i illustrates a physical view of a broad‐spectrum, high‐absorption copper surface used for emissivity testing, with the black structured area measuring 3 cm in length and width, indicating that the method allows for the fabrication of large‐area structures. Confocal microscopy was used to characterize the structure shown in Figure 5i, and its 3D morphology is shown in Figure 5j, where it can be seen that it is homogeneous, and the corresponding cross‐sectional curves are shown in Figure 5k.

It is theoretically expected that the greater the height of the cone‐tip structure, the better the absorption of incident light. Figure 5l presents the results of the emissivity tests in the mid‐infrared band (2.5–16 µm) for the same period, including Figure 5e–h, with different heights of the structure, and it can be clearly observed that when the height of the structure is 30 µm, its emissivity is poorer, but it also reaches more than 80%. When continuing to increase the height of the cone‐tip structure, its emissivity increases, but the increasing trend is slowing down, and finally, an average of 98.6% emissivity in the mid‐infrared band is achieved at a period of 30 µm and a height of 210 µm. Additionally, the test range was extended to the short‐wave and long‐wave regions, respectively, demonstrating the average emissivity of the structure in the 200 nm–25 µm range. As shown in Figure 5m, it can be seen that the fabrication of micro‐ and nanocomposite structures achieves an average of 98% emissivity even in such a wide range of wavelengths spanning the UV‐VIS‐NIR‐MIR. More so, an emissivity of more than 99% was achieved in the wavelength range of 400–700 nm (inset of Figure 5m). In brief, ultra‐broad‐spectrum high‐absorption copper surfaces were realized because of the synergistic effect of the micro‐ and nanocomposite cone‐tip structures. The SEM images of a single nanoparticle plane and the corresponding test results are given in Figure S5 (Supporting Information), where it can be observed that the emissivity is not increased a lot if only the nanoparticles are available.

In addition, it is worth mentioning that the micro‐ and nanocomposite structures fabricated by this strategy have a very uniform emissivity response in the wavelength range of UV‐MIR due to the rational structural design, which achieves a higher and uniform light absorption effect in ultra‐wide spectral range, especially with great potential in applications where it is necessary to maintain consistent emissivities at different wavelengths. And since the strategy is to directly fabricate micro‐ and nanocomposite structures of homologous materials on the surface of the substrate material, which belongs to laser subtractive fabrication, it possesses very excellent stability. As shown in Figure 5n,o, the broad‐spectrum highly absorbing metal surfaces fabricated by the method were tested for stability at a high temperature of 150 °C and a low temperature of minus 196 °C for 24 and 48 h, respectively. The test results showed that the structures were well stabilized. To further test the stability of the structure, we sonicated the machined structures for 1, 2, 3, 4, 5, and 10 h, giving SEM maps and emissivity test results before and after sonication, as in Figure S6 (Supporting Information). After femtosecond laser processing, the surface of the structure is covered with a layer of poorly bound nanoparticles. At this time the structure is sonicated using an ultrasonic cleaner with a power of 80W and an ultrasonic frequency of 40KHz. Figure S6a (Supporting Information) is the SEM image before sonication, and Figure S6b–g (Supporting Information) are the SEM images of sonication for 1, 2, 3, 4, 5, and 10 h, respectively, and it can be clearly observed that after sonication removes the poorly‐bonded nanoparticles from the surface of the structure, there is basically no change in the surface of the structure when it is continued to be sonicated for a longer period. It can also be seen from the emissivity test results in Figure S6h (Supporting Information) that there is a slight decrease in emissivity after sonication compared to when it was not sonicated, but the emissivity remains essentially unchanged by continuing to increase the sonication time. Figure S7 (Supporting Information) compares the emissivity changes of the samples under different environmental factors. The emissivity of the samples under wet environment was compared by fully soaking the samples in deionized water for different times as shown in Figure S7a (Supporting Information).The emissivity of the samples under mechanical pressure was compared by using a standard weight of 1 kg pressed on the samples and kept for different times as shown in Figure S7b (Supporting Information). From the results of the emissivity tests, it can be found that the emissivity of the samples remains essentially unchanged under the environmental factors of wetting and mechanical stress. Lastly, since the “V”‐ scanning method is designed to achieve a broad‐spectrum high absorption effect by rational structural design, the method is also applicable to different metallic materials and even some non‐metallic materials. Figure S8 (Supporting Information) shows the top view of the electron microscope and the results of the emissivity test of micro‐ and nano‐composite structures fabricated on the surface of stainless steel and titanium alloy by using the same method, which proves the no‐material‐selectivity of the method.

## 3 Conclusions

In summary, this paper proposes a method of femtosecond laser “V”‐ scanning and answers the question of what kind of structure can have the best ultra‐wide spectrum high absorption effect through theoretical guidance, verifies the necessity of “V”‐ scanning, and further guides the subsequent fabrication of micro‐ and nanostructures on copper surfaces. The structures consists of a micrometer cone‐tip structure with a depth‐to‐width ratio of 7:1 (period 30 µm, depth 210 µm), an oxide layer with a thickness of more than 2 µm, and nanoparticles of different sizes, achieving uniform high absorption rates exceeding 99% in the localized spectral range of 400–700 nm and over 98% from the UV to MIR range of 200 nm to 25 µm. Moreover, the method applies to different metallic and even non‐metallic materials and possesses excellent high and low‐temperature stability. Overall, through a comprehensive exploration of the light absorption mechanism and experimental methodology of micro‐ and nanostructures, a generalized approach is proposed, which ultimately achieves a truly ultrawide spectrum metallic plane blackbody with high absorption from 0.2–25 µm, and this strategy has great potential in the fields of infrared calibration, passive radiation cooling, and stray light suppression.

## 4 Experimental Section

### Materials and Laser Fabrication

The surface of the copper sample was first wiped using acetone and ethanol, then rinsed with deionized water dried, and set aside. The copper substrate is available in two sizes, the first with length, width, and height of 1 cm, 1 cm, and 1 mm, respectively, for the fabrication of small structures with a length and width of 1 mm and easy characterization of their microscopic morphology and adjustment of laser parameters. The second one has a length, width, and height of 5 cm, 5 cm, and 1 mm, respectively, and is used to fabricate and test the emissivity of a large structure with a length and width of 3 cm. The laser source is a 515 nm femtosecond laser (Light Conversion Pharos) with a repetition frequency of 200 KHz and a pulse width of 280 fs. The combination of an F‐Theta lens and a displacement stage provides a wide range of processing capabilities at high speeds.

### Characterization

The surface and cross‐sectional morphology of the micro‐ and nanocomposite structures were measured by cold field emission scanning electron microscopy (FE‐SEM, JEOL, JSM‐6700F, Japan). The 3D morphology of the micro‐ and nanocomposite structures was measured by laser confocal microscopy (LSCM, OLS4100, Japan), and its cross‐sectional profile was obtained. Elemental distributions on the surface and cross‐section of the micro‐ and nanocomposite structures were characterized by thermal field scanning electron microscopy energy spectroscopy (energy dispersive spectrometer, EDS, Oxford, Ultim Extreme). The chemical state of the surface structure of the samples was measured by a multifunctional scanning X‐ray photoelectron spectroscopy (XPS, TRI, Rigaku Corporation). The diffuse reflectance of the sample in the visible and near‐infrared regions was measured by a UV‐VIS‐NIR spectrophotometer (UV‐VIS‐NIR Spectrometer, UV‐3600i Plus, Shimadzu) equipped with an integrating sphere. The diffuse reflectance of the samples in the NIR‐MIR region was measured by an FTIR Spectrometer (FTIR Spectrometer, INVENIO‐R, Bruker) equipped with an integrating sphere. The emissivity of the sample was obtained from the 1‐diffuse reflectance.

## Conflict of Interest

The authors declare no conflict of interest.

## Supporting information



Supporting Information

## Data Availability

The data that support the findings of this study are available from the corresponding author upon reasonable request.
