# Supplementary material for: Ultrawide Spectrum Metallic Plane Blackbody with Extremely High Absorption from 0.2 to 25 µm
Source: Adv Sci (Weinh). 2024 Nov 21;12(2):2411448. doi: 10.1002/advs.202411448 (PMC11727123; doi:10.1002/advs.202411448)
Supplement: Supplementary file 1 — Supporting Information [file ADVS-12-2411448-s001.docx]

Supporting Information

Ultra-wide spectrum metallic plane blackbody with extremely high absorption from 0.2 μm – 25 μm

Jin-Yong Qi, Xue-Qing Liu*, Zi-Jian Liu, Xin Zhang, Chao Li, Qi-Dai Chen, Lei Wang* and Hong-Bo Sun*

Table S1:

| **Table S1.** Summary of some current work to achieve high absorption properties | | | | |
| --- | --- | --- | --- | --- |
| Material | Method | After treatment | Average absorption | Reference |
| Copper | F-Theta lens |  | 400-750nm: 97.3%  1000-1800nm: 94% | [24] |
| Copper | F-Theta lens |  | 220-800nm: 98.9%  220-2500nm: 91.9% | [25] |
| Copper | F-Theta lens | Thermal oxidation | 14-18μm: about 90%->97%  5-25μm: about 20%->87.5% | [26] |
| Copper | Convex lens | Chemical solution corrosion | 200-380nmn: 96.37%->98.3%  380-780nm: 93.39%->96.06%  780nm-1.5μm: 91.48%->97.22%  1.5μm-16μm: 96.41%->98.93% | [27] |
| 2A12 aluminum alloy | F-Theta lens |  | 400-800nm: 97.4%  400-2500nm: 94.86% | [28] |
| 304L stainless steel | Convex lens |  | 2.5-15μm: 95% | [29] |
| TC4 titanium alloy | F-Theta lens |  | 250-2250nm: 96.9% | [30] |
| N6 nickel | Convex lens |  | 200-400nm: 98%  400-800nm: 97%  800-2000nm: 90% | [31] |
| Silicon | F-Theta lens |  | 300-2500nm: 97.94%  2.5-16μm: 95.02% | [32] |

Figures S1~S8:





**Figure S1.** Schematic and morphology of the structures corresponding to different periods of the conventional linear scanning method. a-b) Too large scanning period. c-d) Large scanning period. e-f) Appropriate scanning period.





**Figure S2.** Modulation of structural depth by conventional linear scanning method. a) Schematic diagram of linear scanning method unfavorable for fabrication of high depth-to-width ratio structures. b) Cross-sectional morphology of the notch obtained by the linear scanning method when varying the scanning speed of the laser. c-f) Enlarged graph of cross sections for four scanning speeds: 5 mm/s, 10 mm/s, 50 mm/s, 100 mm/s. g) Cross section curves corresponding to c-f.





**Figure S3.** a) General overview of the “V”-scanning and b-f) schematic representation of the area removed from the cross-section for different number of scans.





**Figure S4.** FIB test results for the structural cross section of the cone tip.





**Figure S5.** SEM images and emissivity test results of single nanoparticle plane. a-b) Different magnification. c) Emissivity test results.





**Figure S6.** SEM images of the structure before and after ultrasound and the emissivity test results. a) Untreated. b-g) Ultrasound for 1h, 2h, 3h, 4h, 5h and 10h, respectively. h) Emissivity test results for different ultrasound times.





**Figure S7.** Long-term aging test results. a) humidity. B) mechanical stress.





**Figure S8.** Validation of “V”- scanning method for fabrication of micro- and nanocomposite structures on 314L stainless steel (a-b) and TC4 titanium alloy (d-e) surfaces. c) and f) Approximately 98% emissivity test results for both metals in the 2.5-16 μm.
